# European Union Structural Funds as the Source of Financing Health Care Infrastructure Investments in Poland—A Longitudinal Analysis

**DOI:** 10.3389/fpubh.2022.873433

**Published:** 2022-03-25

**Authors:** Katarzyna Dubas-Jakóbczyk, Anna Kozieł

**Affiliations:** ^1^Health Economics and Social Security Department, Institute of Public Health, Faculty of Health Sciences, Jagiellonian University Medical College, Krakow, Poland; ^2^Health, Nutrition and Population, World Bank, The World Bank Office in Poland, Warsaw, Poland

**Keywords:** European Union, structural funds, health care, infrastructure, investment

## Abstract

European Union (EU) structural funds aim at reducing economic and social disparities between the member states regions. The objectives of the study were to (1) provide a summary overview of all health related projects co-financed by EU structural funds in Poland between 2004 and 2020, (2) define the share of projects/funds devoted to infrastructure investments, and (3) assess the total value of EU structural funds' contribution to health care infrastructure investments in comparison to the national public budgets. Data on projects co-financed by EU structural funds covered all projects realized under three financial perspectives: 2004–2006; 2007–2013; 2014–2020. The extraction of “health-related” projects was done according to both the type priority under which the project was realized as well as the type of beneficiary. Results showed that between 2004 and 2020, 14,179 health related projects were implemented, with a total value of 33.2 billion PLN, including EU contribution of 22.8 billion PLN (68.7%). Although projects focused on education and public health prevailed in terms of their total number, infrastructural projects consumed the vast majority of funds. Within the analyzed period, a total of 6,689 health infrastructure projects were implemented with a total value of 29.5 billion PLN, including 19.7 billion PLN of EU contributions. The results confirm that the EU structural funds constituted an important source of infrastructural investments in the Polish health system and majority of them were consumed by investments in public hospitals.

## Introduction

European Union (EU) structural funds aim at reducing economic and social disparities between the member states regions (1). The two major funds include the European Regional Development Fund (ERDF) and the European Social Fund (ESF). Together with other types of funds (e.g., Cohesion Fund) they constitute the main instruments of the EU Cohesion Policy, aimed at strengthening economic, social and territorial cohesion in the European Union (2). These funds finance national and/or regional operational programmes with predefined priorities and project types, as well as beneficiaries eligible for support. The funds are planned, budgeted, and allocated according to financial perspectives. Beginning with the accession to the EU in 2004, Poland benefited from the structural funds under the three financial perspectives of 2004–2006, 2007–2013, and 2014–2020. The general rule of the EU structural funds is the principle of co-financing. The share of the EU contribution in a total project's costs can vary significantly depending on the programme, type of project and/or type of beneficiary. The health sector is only one of many benefiting from the funds. Under the recently completed financial perspective of 2014–2020, Poland was the biggest beneficiary of EU structural funds in terms of both the total allocation, as well as its share devoted to health related projects (for the period 2014–2020, the latter constituted ~3.81% of total EU fund allocation for Poland, in comparison to the average of 2.63% for all EU-28 countries) (3).

Healthcare infrastructure includes both built environments (buildings, transportation terminals/roads) and supporting elements, e.g., medical and non-medical equipment as well as information technology solutions (4). The term “capital investment in health” typically refers to expenditures in construction/renovation of hospitals and other facilities, investment in diagnostic and treatment technologies, and IT platforms. These investments are characterized by their longevity and they are critical to efforts to improve healthcare quality and efficiency (5). In Poland, capital investment is funded separately from health service delivery (which is contracted by and paid for by the public payer—the National Health Fund). For capital investments, public providers (mainly hospitals) usually receive dedicated subsidies from their owners (i.e., local governments) and/or from the state (central) budget. Investments can also be funded from other sources, including external funds and commercial bank loans, as well as charitable donations. Investments in private hospitals are usually funded from the owners' own capital and/or bank loans (6). In the case of both public as well as private health care providers, an important source of external funds for health sector investments in Poland are EU structural funds (7, 8).

Numerous evaluation reports are published for specific programmes and/or priorities co-financed by EU structural funds, including those supervised by the Ministry of Health (9). However, these reports usually focus mostly on the use of funds and input based evaluations, cover a single priority, use a qualitative approach, and/or are based on regional samples. Also, evaluative studies of health-related projects usually focus solely on projects realized under priorities devoted directly to the health sector (e.g., “improving healthcare infrastructure”) and omit those where beneficiaries were also health care stakeholders, yet the projects were realized under some general priorities (e.g., “innovations in enterprises,” “effective public administration,” etc.). As a consequence, no comprehensive, longitudinal overview of all health related, EU co-financed projects is available.

The **objectives of this study** were as follows: (1) to provide a summary overview of all health related projects co-financed by the EU structural funds in Poland between 2004 and 2020; (2) to define the share of projects devoted to infrastructure investments among all health-related projects; and (3) to assess the total value of EU-contribution to health care infrastructure investments in comparison to national public funds (central and local government budgets).

## Methods

Data on projects co-financed by EU structural funds were retrieved from the Ministry of Development Funds and Regional Policy in July 2021 (based on a direct authors' request). The data covered all projects realized under three financial perspectives: 2004–2006; 2007–2013; 2014–2020. The classification of a project as “health-related” was done according to both the type of specific priority/task under which the project was realized (e.g., Priority: investments in health infrastructure) as well as the type of beneficiary (e.g., health services provider, Ministry of Health, Center for Health Information Systems, etc.). All types of health beneficiaries were taken into consideration, regardless of their legal status (private, public). The comparison of data between the three financial perspectives was limited due to differences in the data presentation, thus the summary overview was done by using the operational programme categories (Supplementary Table 1). The type of operational programme roughly defines the type of project; thus the total number and value of projects in the two categories should be treated as a rough approximation and not an exact estimation. The two project categories were defined as follows:

(A) Projects focused on infrastructure investments (investments in buildings, equipment, IT infrastructure as well as research and development, and e-health solutions)—financed from ERDF;
(B) Projects focused on education and public health (educational projects including management and/or IT competencies improvement for health managers, post-graduate training for medical professionals as well as diverse health promotion and disease prevention programmes)—financed from ESF.

For each project, the following information was retrieved: the financial perspective under which the project was realized (years), the name of the operational programme, total value of the project, value of the EU contribution, type of beneficiary, area of the project's realization (a region vs. the whole country).

The total value of the EU contribution to health care infrastructure investments was compared to public budget spending on the same purposes. The budgetary data were retrieved from the Ministry of Finance *via* The Open Budgets Portal operated by the World Bank. The data covered both central budget (CB) and local governments' (LG) budgets expenditures on health between 2004 and 2020. In the case of local governments, the data were summarized for the four types/levels of the local government units: municipalities, cities with a county status, counties, and voivodeships. For both central and LG budgetary data, the following expenditures classification hierarchy was applied: (1) total expenditures, incl., (2) expenditures within Chapter 851 (Health care), incl., (3) capital expenditures, incl., (4) capital expenditures financed *via* a body's own revenue sources. The latter was distinguished to avoid “double-counting,” e.g., when a LG realized an EU co-financed project.

## Results

### General Overview of Health Related Projects Co-financed by EU Structural Funds Between 2004 and 2020

Under the three financial perspectives of 2004–2006, 2007–2013, and 2014–2020 14,179 health related projects were implemented, with a total value of 33.2 billion PLN, including EU contributions of 22.8 billion PLN (68.7%). The majority of the projects were realized and the funds were allocated under the recent financial perspective of 2014–2020 (Table 1). The beneficiaries of health-related projects included health facilities (both public and private); public administration, including central and local government representation; enterprises functioning in the health sector (SMEs), and non-government organizations (NGOs).

**Table 1:** Health related projects co-financed by EU structural funds between 2004 and 2020, per financial perspective, type of operational programme, and project.

**Financial perspective**	Operational programme acronym^*^	Type of projects^**^/main beneficiaries	**No of projects**	**Value of the projects (million PLN)**	**EU contribution (million PLN)**
2004–2006	INTERREG	(A) - Investments in buildings, equipment, IT infrastructure/local governments, and healthcare providers	28	84.62	49.18
	ZPORR	(A) - Investments in buildings, equipment, IT infrastructure/local governments, and healthcare providers	1,095	2,880.42	1,631.09
		**SUM (2004–2006)**	**1,123**	**2,965.04**	**1,680.28**
2007–2013	POIŚ	(A) - Investments in buildings, equipment, IT infrastructure/local governments, healthcare providers, MoH, and medical universities	411	2,392.05	1,873.82
	RPOs (per region)	(A) - Investments in buildings, equipment, IT infrastructure/local governments, and healthcare providers	1,673	6,499.84	4,083.82
	POIG	(A) - IT infrastructure and solutions, research and development/healthcare providers, and SME	45	1,401.29	881.22
	POPT	(A) - Technical support for project realization/MoH	9	28.44	23.92
	POKL	(B) - Education and health promotion/MoH, other government units, medical universities, local governments, NGOs, and SME	373	676.88	570.47
		**SUM (2007–2013)**	**2,511**	**10,998.50**	**7,433.26**
2014-2020	POIŚ	(A) - Investments in buildings, equipment, IT infrastructure/local governments, healthcare providers, MoH, and medical universities	567	4,237.36	2,815.88
	RPOs (per region)	(A) - Investments in buildings, equipment, IT infrastructure/local governments, and healthcare providers	2,553	10,156.12	7,142.24
	RPOs (per region)	(B) - Education, health promotion and disease prevention/local governments, healthcare providers, and NGOs	6,689	2,340.58	1,967.46
	POIR	(A) - IT infrastructure and e-solutions, research and development/healthcare providers, and SME	260	1,203.01	709.04
	POPC	(A) - IT infrastructure, e-solutions/MoH, and other government units	8	437.27	369.74
	POPW	(A) - IT infrastructure and e-solutions, research and development / healthcare providers, and SME	40	224.92	119.75
	POWER	(B) - Education, health promotion and disease prevention/MoH, other government units, medical universities, local governments, NGOs, and SME	428	666.35	572.54
		**SUM (2014–2020)**	**10,545**	**19,265.61**	**13,696.65**
		**TOTAL 2004–2020**	**14,179**	**33,229.14**	**22,810.19**

* *Operational programmes full names: INTERREG, Interregional programmes; ZPORR, Integrated Regional Operational Programme; POIŚ, Operational Programme Infrastructure and Environment; RPO, Regional Operational Programmes; POIG, Operational Programme Innovative Economy; POPT, Operational Programme Technical Support; POKL, Operational Programme Human Capital; POIR, Operational Programme Intelligent Development; POPC, Operational Programme Digital Poland; POPW, Operational Programme Eastern Poland; POWER, Operational Programme Knowledge Education Development*.

** *The two project categories include: (A) infrastructure investments—financed from ERDF; (B) education and public health—financed from ESF*.

**Figure 1:**
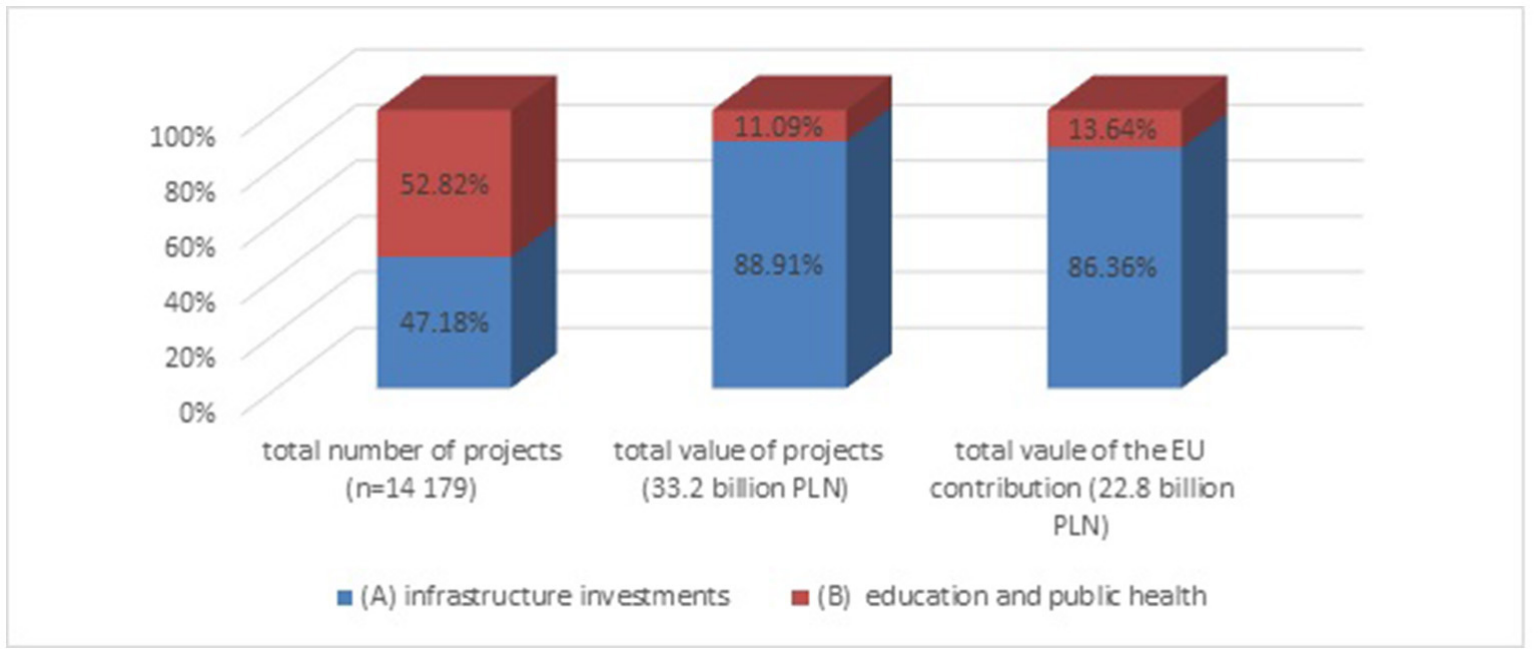
Share of the two types of projects in: the total number of health related projects, their total value, and EU-contribution for health related projects between 2004 and 2020 (%).

In terms of the number of projects: 47.2% of the projects were devoted to infrastructure investments (category A), while 52.8% were devoted to education and public health (category B). Yet, while taking into account the value of the projects (both total and EU contribution), the infrastructure investment projects constituted the vast majority: 88.9% (29.5 billion PLN) of the total value of the projects and 86.4% (19.7 billion PLN) of the EU contributions (Figure 1).

The projects were realized in all 16 voivodships/regions. However, there were also projects whose implementation covered the whole country. The latter included, e.g., several health data digitalization projects run by the Center for E-Health, the MoH or the National Health Fund under the Operational Programme Digital Poland (POPC) 2014–2020, or several educational projects (e.g., post-graduate trainings for medical staff) run by the MoH under the Operational Programme Knowledge Education Development (POKL) 2007–2013. In general, during the two financial perspectives of 2007–2013 and 2014–2020 realization of a total 183 health related projects covered the whole country (with a total value of the projects of 3.3 billion PLN, including 2.4 billion PLN of EU contributions). This included 78 infrastructural projects (total value of 2.4 billion PLN, incl. 1.7 billion PLN of EU contributions) and 105 projects focused on education and public health (total value of 0.9 billion PLN, incl. 0.7 billion PLN of EU contributions).

### Health Infrastructure Projects Co-financed by EU Structural Funds

In the case of projects focused on infrastructure investments (category A)—the majority of the projects were realized directly by healthcare providers (Table 2). Just within the two financial perspectives of 2007–2013 and 2014–2020, a total of 2,671 infrastructure investment projects were realized by public health care providers functioning in the form of independent health care units (*samodzielne publiczne zakłady opieki zdrowotnej—SPZOZs*). SPZOZs, which are the dominant legal form of public hospitals in Poland consumed ~10.3 billion PLN of EU contributions, which constituted more than 57.08% of the total EU contributions for health infrastructural investment projects between 2007 and 2020. However, it can be assumed that the majority of the infrastructural projects run by local governments also focused on public hospitals (as local government units constitute their owners). In summary, between 2007 and 2020 infrastructure investments in public hospitals (realized by both the SPZOZs themselves and their owners—local governments) consumed 12.7 billion PLN of EU contributions (67.54% of the total EU contributions for health infrastructural investment projects and 57.60% of the total EU contributions for all health related projects between 2007 and 2020). These numbers might be underestimated as other types of public hospitals could benefit from EU funds for infrastructure investments (e.g., research institutes supervised by the MoH or University clinics owned by medical universities) classified as an “Other” type of beneficiary in Table 2.

**Table 2:** Infrastructure investments health related projects co-financed by the EU structural fund (ERDF) between 2004 and 2020, per financial perspective and type of beneficiary^*^.

**Financial perspective**	**Type of beneficiary**	**No of projects**	**Value of the projects (million PLN)**	**EU contribution (million PLN)**
2004–2006	Healthcare providers	577	1,049.94	647.06
	Local governments	433	1,015.09	516.77
	Other	113	900.01	516.44
	SUM (2004–2006)	1,123	2,965.04	1,680.28
2007–2013	Public healthcare providers (SPZOZ)	1,457	6,755.35	4,569.08
	Local governments	181	751.33	483.40
	Central government organizational unit	46	853.10	451.06
	Other	454	1,961.84	1,359.25
	SUM (2007–2013)	2,138	10,321.62	6,862.79
2014–2020	Public healthcare providers (SPZOZ)	1,214	8,365.26	5,716.65
	Local governments	129	1,807.88	1,401.97
	Central government organizational unit	14	1,328.95	1,078.32
	Other	2,071	4,756.59	2,959.71
	SUM (2014–2020)	3,428	16,258.68	11,156.65
	**TOTAL (2004–2020)**	**6,689**	**29,545.33**	**19,699.71**

* *For the period 2004–2006 no information was available on the type of healthcare provider; for the two following financial perspectives it was possible to extract the projects realized by public health care providers (SPZOZ) while different types of healthcare providers, including those functioning in a form of NGOs and/or SMEs are covered by the “Other” beneficiary category*.

### Comparing the Value of EU Contributions to the National Budgets' Capital Expenditures on Health

Both central and local governments devote a relatively small share of their total budgets to health: 2.27 and 1.96%, respectively (the average between 2004 and 2020). On average, the capital expenditures on health constitute ~15.09% of the total health expenditures in the case of the central budget and 40.76% in the case of local governments (Supplementary Tables 2, 3). Yet, in case of the latter there are differences between the four types/levels of LG units (Supplementary Tables 4–7). The average share of capital expenditures in total health care spending varied in the analyzed period from: 18.86% in municipalities to as much as 69.86% in voivodships.

**Figure 2:**
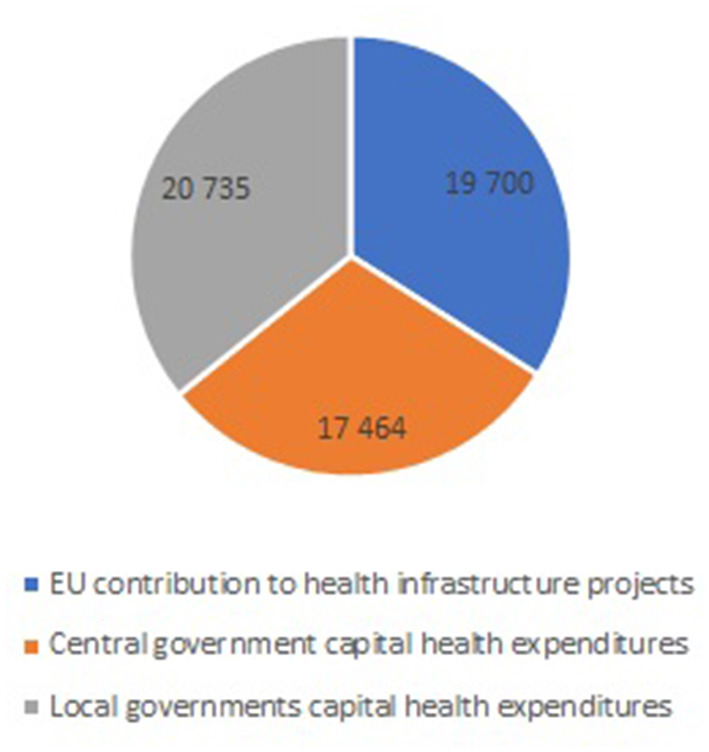
The share of the three sources in total capital expenditures on health between 2004 and 2020 (Million PLN).

In general, the total value of capital health expenditures financed from their own revenues between 2004 and 2020 was ~17.5 billion PLN for central and 20.7 billion PLN for local governments. As a consequence, the total capital expenditures on health financed from the EU structural funds and public budgets between 2004 and 2020 amounts to 57.9 billion PLN. The share of the three sources is as follows: 34.02% is the EU contribution to infrastructural projects; 30.16% constitute central budget expenditures while the remaining 35.81% constitute local government expenditures (Figure 2).

On the average, the total capital expenditures on health financed from the EU structural funds and public budgets constituted in the analyzed period ~3.02% of the total current health expenditures and 0.22% of the gross domestic product (Supplementary Table 8).

## Discussion

The study provides a comprehensive overview of the EU structural fund's role in financing health related projects in Poland since the country's accession to the EU. Between 2004 and 2020, more than 14,000 health related projects were implemented, with a total value of 33.2 billion PLN, including EU contributions of 22.8 billion PLN. A total of 6,689 health infrastructure projects were implemented with a value of 29.5 billion PLN, including 19.7 billion PLN of EU contributions (86.36% of the total EU contributions to health related projects between 2004 and 2020).

The results showed that between 2007 and 2020, infrastructural investment in public hospitals consumed the majority of the total EU contributions for health related projects. Poland is characterized by oversized hospital infrastructure. The number of hospital beds (both total and acute) per 1,000 population is above the EU average, and inpatient care spending accounts for the majority of health expenditures (10). At the same time, the existing hospital units are in constant need of new infrastructural investments (8). Although, numerous analyses indicate the problem of overcapacities in the hospital sector in Poland with simultaneous deficits in primary, specialist ambulatory and long-term care (8, 10), no major changes have been observed within the last 15 years. For example the number of hospital beds per 1,000 population decreased only slightly from 6.7 in 2004 to 6.2 in 2019 (11).

The comparison of data between the three financial perspectives is limited due to differences in data presentation. Yet, the general overview shows that the majority of projects were realized and funds were allocated under the recent financial perspective of 2014–2020. This was also a perspective in which, for infrastructure projects, new rules of application assessment and granting financing were introduced, including developing the health needs maps and a tool for assessing investment proposals—the Evaluation Instrument of Investment Motions in Health Care. The latter aimed at improving capital investment coordination, i.e., by limiting duplication of investment in the same geographical area and assuring that the planned investment is actually needed (in accordance with the regional health needs map) (12).

Our results confirm that the EU structural funds constitute an important source of infrastructural investments in the Polish health system. In the analyzed period, the total value of EU structural fund contributions (19.7 billion PLN) was comparable to the total capital health expenditure from central budgets (17.5 billion PLN) as well as the sum of local government budgets (20.7 billion PLN). These results are in line with available fragmented data/estimations from international statistics. According to the OECD data, among the EU-27 countries, capital expenditures on health constitute on average 0.4% of the GDP and 5.0% of current health expenditures. In general, levels of capital investment on health vary significantly both across countries and over time, even more so than overall health spending (13). In the case of Poland, the data for 2018 cover both the health and social care sectors and show that capital expenditures on health constituted 7.1% of current health expenditures and 0.4% of the gross domestic product. The archive data show that in 2013, ~56% of the total capital expenditures on health were public (14). Our estimations showed that in the analyzed period, the total capital health expenditures, limited to those financed from public budgets and EU structural funds, was at the average level of: 3.02% of the total current health expenditures and 0.22% of the gross domestic product.

The literature provides mixed evidence on the association between healthcare infrastructure and the quality of care or health outcomes (15, 16). In general, it is difficult to directly connect health infrastructure investments with final health outcomes or even health care utilization indicators as numerous other variables influence the latter: e.g., on the supply side (medical professionals' availability), on the demand side (age structure of the population, physical distance to providers), and influencing both sides—providers' payment methods (expenditures/contracts limits, co-payment principles). In the Polish context, an additional challenge is the limited data availability. The evaluation reports prepared for the specific EU programmes' priorities usually include some infrastructure capacity indicators as well as qualitative assessment based on the direct beneficiary institution's representatives' opinions. For example, the mid-term evaluation report of Priority no IX ‘Supporting strategic health care infrastructure' of the Operational Programme Infrastructure and Environment 2014–2020, covered indicators related to both “raw” infrastructure (e.g., the number of providers receiving support, the number of renovated buildings or equipment bought) and its utilization (e.g., the number of patients treated). The assessment of the realized project's impact on the quality of care was done based on the subjective opinions of the representatives of the beneficiary institutions (e.g., *via* focus groups with hospital managers or dedicated questionnaires) (17). As a consequence, future research should focus on developing the methodology and conducting a more comprehensive impact evaluation of the capital investments in health.

There are several limitations to be noted. First of all, the statistics concerning the number and value of projects should be treated with caution. As mentioned in the methods section, the comparison of the data between the three financial perspectives is limited. A significant number of projects identified between 2014 and 2020 might be related to a more detailed data availability for this period than the previous perspectives. Secondly, the estimations related to the value of total capital investments cover only EU-structural funds and public budgets. Thus, this omits private sources as well as other external ones, e.g., the European Economic Area Financial Mechanism and the Norwegian Financial Mechanism (so called Norway grants), the Swiss-Polish Cooperation Programme (so called Swiss Contribution funds), and capital investments realized under EU research and innovation programmes like the 7th Framework Programme 2007–2014, Horizon 2020, or EU Health Programmes. As a consequence, our results provide rather rough estimations and not exact calculations and focus on a limited number of external sources of financing health care infrastructure investment.

## Data Availability Statement

The original contributions presented in the study are included in the article/Supplementary Material, further inquiries can be directed to the corresponding author.

## Author Contributions

KD-J and AK: conceptualization, methodology, acquisition of data, manuscript review, and revision. KD-J: data analysis, findings interpretation, and manuscript writing. Both authors meet the authorship criteria and agree to the submission of the manuscript and have made substantial contributions to the conception or design of the work, according to the International Committee of Medical Journal Editors (ICMJE), and to the Committee on Publication Ethics (COPE).

## Funding

The World Bank provided financial support for the preparation of the Report: “The infrastructure investments on health in Poland between 2004 and 2020—a quantitative analysis” and holds the copyright to it.

## Author Disclaimer

The authors are solely responsible for their views presented in this article and they do not necessarily express the views or policies of the World Bank. The World Bank does not guarantee the accuracy of the data included in this work.

## Conflict of Interest

The authors declare that the research was conducted in the absence of any commercial or financial relationships that could be construed as a potential conflict of interest.

## Publisher's Note

All claims expressed in this article are solely those of the authors and do not necessarily represent those of their affiliated organizations, or those of the publisher, the editors and the reviewers. Any product that may be evaluated in this article, or claim that may be made by its manufacturer, is not guaranteed or endorsed by the publisher.
