# Supplementary material for: European Union Structural Funds as the Source of Financing Health Care Infrastructure Investments in Poland—A Longitudinal Analysis
Source: Front Public Health. 2022 Mar 25;10:873433. doi: 10.3389/fpubh.2022.873433 (PMC8989962; doi:10.3389/fpubh.2022.873433)
Supplement: Supplementary file 1 [file Table_1.pdf]

Supplementary Material

Supplementary tables

Table 1. Main operational programs in Poland, financed by the EU Structural Funds, per financial perspective

Financial perspective	Acronym	Original name	English translation	EU Structural Fund	Health related projects category*
2004-2006	INTERREG	INTERREG Polska-Meklemburgia,; Polska-Brandemburgia; Polska-Saksonia etc.	Interregional programs (Polish – and cross border regions)	ERDF	(A)
	ZPORR	Zintegrowany Program Operacyjny Rozwoju Regionalnego	Integrated Regional Operational Programme	ERDF	(A)
2007-2013	POIŚ	Program Operacyjny Infrastruktura i Środowisko	Operational Programme Infrastructure and Environment	ERDF	(A)
	RPO	Regionalne Programy Operacyjne (per województwo)	Regional Operational Programmes (per each voivodeship)	ERDF	(A)
	POIG	Program Operacyjny Innowacyjna Gospodarka	Operational Programme Innovative Economy	ERDF	(A)
	POPT	Program Operacyjny Pomoc Techniczna	Operational Programme Technical Support	ERDF	(A)
	POKL	Program Operacyjny Kapitał Ludzki	Operational Programme Human Capital	ESF	(B)
2014-2020	POIŚ	Program Operacyjny Infrastruktura i Środowisko	Operational Programme Infrastructure and Environment	ERDF	(A)
	RPO	Regionalne Programy Operacyjne (per województwo)	Regional Operational Programmes (per each voivodeship)	ERDF/ESF	(A) or (B)
	POIR	Program Operacyjny Inteligentny Rozwój	Operational Programme Intelligent Development	ERDF	(A)
	POPC	Program Operacyjny Polska Cyfrowa	Operational Programme Digital Poland	ERDF	(A)
	POPW	Program Operacyjny Polska Wschodnia	Operational Programme Eastern Poland	ERDF	(A)
	POWER	Program Operacyjny Wiedza Edukacja Rozwój	Operational Programme Knowledge Education Development	ESF	(B)

* The two project categories include: (A) projects focused on infrastructure investments (investments in buildings, equipment, IT infrastructure as well research & development, e-health solutions) – financed from ERDF; (B) education and public health (diverse health promotion and disease prevention programs) – financed from ESF.

Table 2. Central budget health expenditures between 2004 and 2020, Million PLN

Column	(0)	(1)	(2)	(3)	Share (%)		
Year / Budget category	Total budget	Incl. health exp.	Incl. capital health exp.	Incl. capital health exp. financed from own revenues	share of (1) in (0)	share of (2) in (1)	share of (3) in (2)
2004	193 020	3 621	898	895	1.88%	24.80%	99.67%
2005	203 771	3 575	880	868	1.75%	24.61%	98.64%
2006	220 250	3 653	837	825	1.66%	22.92%	98.51%
2007	249 655	5 239	1 003	1 001	2.10%	19.14%	99.76%
2008	273 986	6 466	1 273	1 270	2.36%	19.69%	99.77%
2009	298 028	6 772	1 043	1 042	2.27%	15.40%	99.91%
2010	294 894	6 864	1 014	1 014	2.33%	14.77%	100.00%
2011	302 682	7 492	1 055	1 055	2.48%	14.08%	100.00%
2012	318 002	7 242	966	966	2.28%	13.35%	100.00%
2013	321 345	7 534	886	886	2.34%	11.77%	100.00%
2014	312 523	7 358	864	864	2.35%	11.74%	100.00%
2015	331 743	7 325	1 111	1 061	2.21%	15.17%	95.51%
2016	360 093	8 234	1 153	1 142	2.29%	14.01%	98.97%
2017	374 472	9 598	1 738	1 716	2.56%	18.10%	98.74%
2018	385 337	10 479	1 159	1 063	2.72%	11.06%	91.75%
2019	414 135	10 897	1 185	1 088	2.63%	10.88%	91.78%
2020*	369 852	6 323	836	707	1.71%	13.22%	84.61%
**SUM**	**5 223 787**	**118 671**	**17 903**	**17 464**	**2.27%**	**15.09%**	**97.55%**

*data for 3 quarters

Source: Ministry of Finance

Table 3. Local governments (sum for all types of local governments) health expenditures between 2004 and 2020, Million PLN

Column	(0)	(1)	(2)	(3)	Share (%)		
**Year / Budget category**	**Total budget**	**Incl. health exp.**	**Incl. capital health exp.**	**Incl. capital health exp. financed from own revenues**	**share of (1) in (0)**	**share of (2) in (1)**	**share of (3) in (2)**
2004	91 387	2 108	733	726	2.31%	34.79%	98.95%
2005	103 807	2 582	1 076	1 024	2.49%	41.68%	95.14%
2006	120 039	3 067	1 459	1 359	2.55%	47.59%	93.12%
2007	129 113	3 062	1 581	1 523	2.37%	51.63%	96.31%
2008	145 183	3 681	1 965	1 907	2.54%	53.39%	97.07%
2009	167 828	4 105	2 155	1 588	2.45%	52.50%	73.71%
2010	177 766	3 962	1 616	1 344	2.23%	40.79%	83.15%
2011	181 595	4 090	1 346	1 085	2.25%	32.91%	80.63%
2012	180 459	3 515	1 221	1 038	1.95%	34.74%	85.00%
2013	183 839	3 855	957	825	2.10%	24.84%	86.14%
2014	196 754	3 676	1 068	865**	1.87%	29.06%	81.02%
2015	196 415	3 907	1 458	1 070	1.99%	37.33%	73.34%
2016	206 035	3 320	1 117	1 017	1.61%	33.65%	91.02%
2017	230 166	3 479	1 317	1 249	1.51%	37.85%	94.84%
2018	259 386	3 841	1 767	1 540	1.48%	45.99%	87.17%
2019	280 209	4 508	2 278	1 732	1.61%	50.53%	76.02%
2020*	224 475	3 410	1 409	844	1.52%	41.32%	59.93%
**SUM**	**3 074 456**	**60 165**	**24 524**	**20 735**	**1.96%**	**40.76%**	**84.55%**

*data for 3 quarters; **break in the data series thus the amount estimated based on the whole period average
Source: Ministry of Finance

Table 4. Municipalities health expenditures between 2004 and 2020. Million PLN

**Column**	**(0)**	**(1)**	**(2)**	**(3)**	**Share (%)**		
**Year / Budget category**	**Total budget**	**Incl. health exp.**	**Incl. capital health exp.**	**Incl. capital health exp. financed from own revenues**	**share of (1) in (0)**	**share of (2) in (1)**	**share of (3) in (2)**
2004	40 942	373	60	60	0.91%	16.19%	99.63%
2005	45 837	408	83	76	0.89%	20.31%	92.32%
2006	53 180	443	100	83	0.83%	22.60%	82.63%
2007	56 074	450	90	83	0.80%	19.98%	92.70%
2008	62 893	501	112	108	0.80%	22.29%	96.37%
2009	70 003	528	122	109	0.75%	23.16%	89.11%
2010	79 741	563	125	94	0.71%	22.29%	75.10%
2011	79 687	606	91	71	0.76%	14.98%	78.51%
2012	78 491	536	90	77	0.68%	16.81%	85.36%
2013	79 443	542	71	67	0.68%	13.20%	94.23%
2014	85 070	561	82	60**	0.66%	14.65%	72.80%
2015	85 944	553	65	63	0.64%	11.82%	96.90%
2016	98 175	586	90	89	0.60%	15.30%	99.35%
2017	111 984	618	108	104	0.55%	17.55%	95.81%
2018	127 133	668	137	117	0.53%	20.52%	85.04%
2019	135 317	727	171	99	0.54%	23.56%	57.84%
2020*	107 878	456	121	72	0.42%	26.53%	59.60%
**SUM**	**1 397 791**	**9 120**	**1 720**	**1 433**	**0.65%**	**18.86%**	**83.31%**

*data for 3 quarters. ** break in the data series thus the amount estimated based on the whole period average
Source: Ministry of Finance

Table 5. Counties (powiats) health expenditures between 2004 and 2020. Million PLN

Column	(0)	(1)	(2)	(3)	Share (%)		
Year / Budget category	Total budget	Incl. health exp.	Incl. capital health exp.	Incl. capital health exp. financed from own revenues	share of (1) in (0)	share of (2) in (1)	share of (3) in (2)
2004	12 445	605	235	234	4.87%	38.73%	99.84%
2005	13 891	641	247	226	4.61%	38.51%	91.47%
2006	15 593	805	365	314	5.16%	45.34%	86.17%
2007	16 070	719	330	305	4.48%	45.91%	92.30%
2008	18 115	1 036	533	520	5.72%	51.47%	97.53%
2009	21 156	1 173	389	330	5.55%	33.17%	84.83%
2010	23 826	1 395	357	227	5.85%	25.56%	63.56%
2011	24 059	1 517	256	190	6.30%	16.91%	73.94%
2012	22 477	1 149	179	132	5.11%	15.57%	73.85%
2013	22 917	1 327	157	135	5.79%	11.84%	85.84%
2014	23 784	1 274	187	182	5.36%	14.68%	97.33%
2015	23 444	1 184	216	167	5.05%	18.24%	77.40%
2016	23 301	1 036	232	221	4.45%	22.38%	95.15%
2017	25 360	931	267	253	3.67%	28.65%	94.69%
2018	28 657	1 033	384	329	3.61%	37.13%	85.73%
2019	29 631	1 069	462	374	3.61%	43.22%	80.90%
2020*	24 235	741	260	221	3.06%	35.01%	85.05%
**SUM**	**368 959**	**17 635**	**5 034**	**4 358**	**4.78%**	**28.54%**	**86.57%**

*data for 3 quarters.
Source: Ministry of Finance

Table 6. Cities with county status (MNPP) health expenditures between 2004 and 2020. Million PLN

Column	(0)	(1)	(2)	(3)	Share (%)		
**Year / Budget category**	**Total budget**	**Incl. health exp.**	**Incl. capital health exp.**	**Incl. capital health exp. financed from own revenues**	**share of (1) in (0)**	**share of (2) in (1)**	**share of (3) in (2)**
2004	32 137	607	181	181	1.89%	29.87%	100.00%
2005	36 491	667	245	242	1.83%	36.69%	99.05%
2006	41 237	785	322	308	1.90%	41.00%	95.69%
2007	45 877	812	400	390	1.77%	49.26%	97.43%
2008	51 172	909	440	425	1.78%	48.40%	96.63%
2009	56 201	976	414	398	1.74%	42.45%	96.03%
2010	58 954	1 062	424	413	1.80%	39.91%	97.38%
2011	61 510	983	285	261	1.60%	28.98%	91.65%
2012	63 731	879	194	188	1.38%	22.07%	97.19%
2013	64 953	1 058	183	181	1.63%	17.26%	99.17%
2014	69 661	1 065	318	222**	1.53%	29.89%	69.81%
2015	69 824	1 063	350	340	1.52%	32.91%	97.23%
2016	71 942	965	270	259	1.34%	27.98%	95.75%
2017	78 504	1 066	367	355	1.36%	34.41%	96.70%
2018	87 015	1 149	487	468	1.32%	42.36%	96.08%
2019	96 885	1 169	488	438	1.21%	41.74%	89.79%
2020*	77 671	849	286	253	1.09%	33.68%	88.61%
**SUM**	**1 063 766**	**16 064**	**5 653**	**5323**	**1.51%**	**35.19%**	**94.16%**

*data for 3 quarters. ** break in the data series thus the amount estimated based on the whole period average
Source: Ministry of Finance

Table 7. Voivodeships health expenditures between 2004 and 2020. Million PLN

Column	(0)	(1)	(2)	(3)	Share (%)		
**Year / Budget category**	**Total budget**	**Incl. health exp.**	**Incl. capital health exp.**	**Incl. capital health exp. financed from own revenues**	**share of (1) in (0)**	**share of (2) in (1)**	**share of (3) in (2)**
2004	5 864	521	257	250	8.88%	49.30%	97.24%
2005	7 588	866	502	479	11.42%	57.94%	95.50%
2006	10 029	1 034	673	654	10.31%	65.07%	97.21%
2007	11 092	1 080	761	745	9.73%	70.45%	97.89%
2008	13 003	1 234	880	854	9.49%	71.31%	97.09%
2009	20 469	1 427	1 229	751	6.97%	86.15%	61.12%
2010	15 245	943	711	610	6.18%	75.37%	85.91%
2011	16 339	984	714	563	6.02%	72.55%	78.90%
2012	15 761	951	758	640	6.03%	79.70%	84.46%
2013	16 527	927	546	441	5.61%	58.89%	80.81%
2014	18 238	775	501	401**	4.25%	64.66%	80.10%
2015	17 203	1 107	827	499	6.43%	74.75%	60.32%
2016	12 617	732	526	448	5.81%	71.75%	85.33%
2017	14 317	864	575	538	6.03%	66.56%	93.55%
2018	16 582	990	759	627	5.97%	76.64%	82.57%
2019	18 376	1 542	1 156	821	8.39%	74.99%	70.96%
2020*	14 780	1 352	731	287	9.15%	54.09%	39.25%
**SUM**	**244 029**	**17 329**	**12 105**	**9 609**	**7.10%**	**69.86%**	**79.38%**

*data for 3 quarters. ** break in the data series thus the amount estimated based on the whole period average
Source: Ministry of Finance

Table 8. Share of capital health expenditures in total health expenditures, NHF (public payer) budget and GDP between 2004 and 2020. Values in Million PLN.

Column	(0)	(1)	(2)	(3)	Share (%)		
**Year / Category**	**Total capital health exp. (EU + national budgets)**	**Total current health exp.***	**NHF exp. on health services**	**GDP**	**share of (0) in (1)**	**share of (0) in (2)**	**share of (0) in (3)**
2004	2 181	n/a	30 514	933 062	n/a	7.15%	0.23%
2005	2 452	n/a	33 097	990 468	n/a	7.41%	0.25%
2006	2 744	n/a	35 957	1 069 824	n/a	7.63%	0.26%
2007	3 504	n/a	39 037	1 187 605	n/a	8.97%	0.30%
2008	4 158	n/a	51 360	1 286 069	n/a	8.10%	0.32%
2009	3 611	n/a	55 281	1 372 208	n/a	6.53%	0.26%
2010	3 338	92 775	56 821	1 445 297	3.60%	5.87%	0.23%
2011	3 121	97 673	57 979	1 566 813	3.20%	5.38%	0.20%
2012	2 985	101 042	61 685	1 629 392	2.95%	4.84%	0.18%
2013	2 692	105 635	62 742	1 656 842	2.55%	4.29%	0.16%
2014	3 323	107 458	63 793	1 720 430	3.09%	5.21%	0.19%
2015	3 725	114 142	67 880	1 800 228	3.26%	5.49%	0.21%
2016	3 752	121 107	70 793	1 861 112	3.10%	5.30%	0.20%
2017	4 558	130 536	77 064	1 988 730	3.49%	5.91%	0.23%
2018	4 197	134 244	81 121	2 121 555	3.13%	5.17%	0.20%
2019	4 413	147 839	90 343	2 287 739	2.99%	4.89%	0.19%
2020	3 146	165 672	101 360	2 317 089	1.90%	3.10%	0.14%
**Average**	**n/a**	**n/a**	**n/a**	**n/a**	**3.02%**	**5.96%**	**0.22%**

*total current health expenditures (based on System of Health Accounts data)
